# When gold standards are not so golden: prevalence bias in randomized trials on endoscopic colorectal cancer screening

**DOI:** 10.1007/s10654-023-01031-2

**Published:** 2023-08-02

**Authors:** Hermann Brenner, Thomas Heisser, Rafael Cardoso, Michael Hoffmeister

**Affiliations:** 1 Division of Clinical Epidemiology and Aging Research, German Cancer Research Center (DKFZ), INF 581, Heidelberg, 69120 Germany; 2 Division of Preventive Oncology, German Cancer Research Center (DKFZ) and National Center for Tumour Diseases (NCT), Heidelberg, Germany; 3 German Cancer Consortium (DKTK), German Cancer Research Center (DKFZ), Heidelberg, Germany; 4 Medical Faculty Heidelberg, Heidelberg University, Heidelberg, Germany

**Keywords:** Bias, Colorectal cancer, Endoscopy, Incidence, Prevalence, Trial

## Abstract

Randomized trials on the effectiveness of screening endoscopy in reducing colorectal cancer (CRC) risk have reported statistically significant, but rather modest reduction of CRC risk by the screening offer. However, risk estimates in these trials included substantial proportions of prevalent CRC cases which were early detected, but could not possibly have been prevented by screening. Thereby, a key principle of randomized prevention trials is violated that only “at risk” persons who do not yet have the disease one aims to prevent should be included in measures of preventive effects. Using recently published data from the Nordic-European Initiative on Colorectal Cancer (NordICC) trial as an example, we illustrate that approaches aimed to account for “prevalence bias” lead to effect estimates that are substantially larger than those reported in the trial and more in line with results from observational studies and real life settings. More rigorous methodological work is needed to develop effective and user-friendly tools to prevent or adjust for prevalence bias in future screening studies.

## Introduction

Randomized controlled trials (RCTs) are the gold standard approach to establish causality of preventive or therapeutic effects of medical interventions. A commonly employed endpoint for studies of preventive measures is the incidence of the disease one aims to prevent. In such studies, people who already have the disease should be excluded as the intervention can no longer prevent it. Obviously, this important prerequisite has not been fulfilled in the RCT-based estimates of the preventive effects of screening colonoscopy or sigmoidoscopy [1–6], in which a large proportion of presumedly incident colorectal cancer (CRC) cases were already present at baseline. The obvious dilemma here is that identifying prevalent cases at baseline would have required a thorough large bowel exam, such as colonoscopy, among all participants, i.e. the very exam whose efficacy in preventing CRC by removing precancerous lesions would be the subject of investigation. In theory, in such a setting, the effectiveness of CRC prevention could still be assessed in a randomized design in which participants with findings of prevalent CRC at colonoscopy would be excluded and the remaining participants would be randomized in such a way that precancerous lesions would be removed in the intervention group only but not in the control group. Obviously, such an approach would be unethical and not be a viable option.

However, simply including prevalent cases in both the intervention and the control group and not accounting for the resulting bias in estimates of incidence reduction is not a good solution either, as it may lead to strongly misleading results. We use the recently reported first RCT estimates of screening colonoscopy effects on CRC incidence from the Nordic-European Initiative on Colorectal Cancer (NordICC) trial [1] as an example to illustrate this prevalence bias.

## Methods

In the NordICC trial, 84,585 participants aged 55–64 years from Poland, Norway and Sweden were randomly assigned in a 1:2 ratio to the offer of a single screening colonoscopy or usual care [1]. The offer was used by 42% of participants in the screening group. After 10 years of follow-up, the estimated reduction of CRC risk was 18% in intention-to-screen (ITS) analysis and 31% in per-protocol (PP) analysis, respectively.

The prevalence of cancers at recruitment was only known from participants who actually underwent screening colonoscopy. In the NordICC trial, 62 of 102 cancers (61%) observed within 10 years among 11,843 screened participants were already present and detected at screening colonoscopy, i.e., prevalence at screening was 62/11,843 = 0.52%. The baseline prevalence of CRC among the unscreened participants is unknown but the overall prevalence in the invited group and the usual-care group should have been approximately equal, given the randomization and the large sample size. It is therefore plausible to assume identical CRC prevalences in the invited group and the usual-care group. However, selective use of the screening offer might have led to some variation in prevalence between users and nonusers of screening within the invited group, and overall within-group prevalence could therefore be higher or lower than the observed 0.52%. To account for this, we assumed a prevalence of 0.52% in both the intervention group and the usual-care group in our base-case exemplary calculations, and additionally conducted sensitivity analyses assuming a range of theoretically possible and plausible baseline prevalences.

Derivation of the minimum and maximum theoretically possible prevalence is illustrated in Table 1. They were obtained by assuming that all observed CRC cases in the unscreened subgroup of the invited group (n = 157) were either truly incident or prevalent cases. While neither of these extreme scenarios is realistic, true prevalence in the invited group must have been somewhere between the resulting prevalence estimates, i.e. 0.22% and 0.78%.

**Table 1:** Reported CRC cases in the NordICC study, minimum and maximum theoretically possible baseline prevalence of CRC in the invited group

Scenario	Screened *N = 11,843*			Unscreened *N = 16,377*			Total *N = 28,220*
	Prevalent	Incident	Total	Prevalent	Incident	Total	Prevalent
Observed	62 (0.52%)	40	102	unknown	unknown	157	unknown
T_min_	62 (0.52%)	40	102	0	157	157	62 (0.22%)
T_max_	62 (0.52%)	40	102	157	0	157	219 (0.78%)

CRC, colorectal cancer; T_min_ and T_max_, theoretical minimum and maximum of CRC prevalence, respectively

To further narrow down the prevalence estimates to a plausible range, we derived expected prevalence from reported cancer incidence data in 2009–2014, the recruitment period, in the three countries [7, 8], and previously derived estimates of mean sojourn time (MST) of CRC in preclinical phase (which ranged from 3 to 6 years) [9–11]. Let I, P, and T be the annual incidence, (preclinical) prevalence and annual clinical manifestation rate of preclinical CRC. Then incidence and prevalence can be expressed as.

I = P × T, and

P = I / T = I × MST,

Derivation of a plausible range of prevalences using this approach and taking country-, sex- and age-specific incidence rates and shares of the trial population into account is illustrated in Table 2 and yielded prevalence values between 0.29% and 0.58%.

**Table 2:** Expected CRC prevalence in the NordICC trial population derived from sex- and age-specific CRC incidence rates during the recruitment period^a^ and estimates of mean CRC sojourn time from previous studies [9–11]

Metric		Country		
		Poland	Norway	Sweden
Trial population	Men	27,198	13,217	1,984
	Women	27,330	13,194	1,662
CRC incidence age 55–64 (per 100,000)	Men	108.4	133.3	93,2
	Women	66.6	103.0	73,6
		All countries and both sexes combined		
Weighted average CRC incidence^b^		96.9 per 100,000		
CRC prevalence if MST = 3 years		0.29%		
CRC prevalence if MST = 4 years		0.39%		
CRC prevalence if MST = 5 years		0.48%		
CRC prevalence if MST = 6 years		0.58%		

CRC, colorectal cancer; MST, mean sojourn time

^a^ For Poland, CRC incidence rates were extracted for the years 2009–2013 from the European Cancer Information System (ECIS) database [7] (ECIS database did not include data for 2014]; for Norway and Sweden, CRC incidence rates were extracted for the years 2009–2014 from the Nordcan database [8]

^b^ weighted according to the country, sex and age distribution of the NordICC trial study population

We derived ranges of theoretically possible and plausible values of cumulative incidence of truly incident cases by subtracting the so-derived prevalences from the cumulative incidence metrics reported in the NordICC trial, which had included the prevalent cases, and we derived ranges of theoretically possible and plausible values of “prevalence-corrected” risk ratios for truly incident cases obtained after these subtractions for both the ITS and the PP analysis.

## Results

Table 3 shows the reported results of the NordICC trial with the inclusion of cancers that were already present at baseline and the estimated results with the exclusion of prevalent cancers. Reported cumulative incidence of the ITS analysis was 0.98% for the intervention group and 1.20% for the usual-care group, resulting in a risk ratio of 0.82 which corresponds to a risk reduction by 18%. Reported cumulative incidence of the PP analysis was 0.84% for the intervention group and 1.22% for the usual-care group, resulting in a risk ratio of 0.69 which corresponds to a risk reduction by 31%.

**Table 3:** Reported estimates of CRC risk reduction with inclusion of prevalent cases and estimated risk reduction after excluding prevalent cases in the NordICC trial on screening colonoscopy

Analysis	Scenario	Handling of prevalent cases (assumed prevalence)	Cumulative Incidence [%]		Risk ratio	Risk reduction
			Intervention Group	Usual-Care Group		
ITS	**Reported**	**Included** ^**a**^	**0.98**	**1.20**	**0.82**	**18%**
	T_Min_	Excluded (0.22%)	0.76	0.98	0.78	22%
	P_Low_	Excluded (0.29%)	0.69	0.88	0.75	25%
	**Base-case**	**Excluded (0.52%)** ^**b**^	**0.46**	**0.68**	**0.68**	**32%**
	P_High_	Excluded (0.58%)	0.40	0.62	0.65	35%
	T_Max_	Excluded (0.78%)	0.20	0.42	0.48	52%
PP	**Reported**	**Included** ^**a**^	**0.84**	**1.22**	**0.69**	**31%**
	T_Min_	Excluded (0.22%)	0.62	1.00	0.62	38%
	P_Low_	Excluded (0.29%)	0.55	0.93	0.59	41%
	**Base-case**	**Excluded (0.52%)** ^**b**^	**0.32**	**0.70**	**0.46**	**54%**
	P_High_	Excluded (0.58%)	0.26	0.64	0.41	59%
	T_Max_	Excluded (0.78%)	0.06	0.44	0.14	86%

CRC, colorectal cancer; ITS, intention-to-screen; P_Low_ and P_High_, lower and upper end of plausible range of CRC prevalence based on a weighted average of incidence rates in the contributing countries and range of mean CRC sojourn time estimates; PP, per-protocol, T_min_ and T_max_, theoretical minimum and maximum of CRC prevalence, respectively

^a^ as reported by Bretthauer et al. [1]

^b^ base-case analysis assuming equal CRC prevalence in screened and unscreened participants

If prevalent cancers were excluded, the cumulative incidences in the intervention group and the usual-care group would decrease to 0.46 and 0.68, respectively, in the base-case ITS analysis, resulting in a risk ratio of 0.68, i.e. the estimated risk reduction would increase from 18 to 32%. The theoretically possible range of prevalence-corrected risk reduction would be from 22 to 52%, and a plausible range of risk reduction derived from cancer-registry data would be from 25 to 35%.

In the PP analysis, base-case exclusion of 0.52% prevalent cases would lead to cumulative incidences of 0.32% and 0.70% in the intervention group and the usual-care group, respectively, resulting in a risk ratio of 0.46, i.e. the estimated risk reduction would increase from 31 to 54%. The theoretically possible range of prevalence-corrected risk reduction would be from 38 to 86%, and a plausible range of risk reduction derived from cancer-registry data would be from 41 to 59%.

## Discussion

The exemplary calculations based on published results from the NordICC trial provided in this article suggest a much stronger preventive effect of screening colonoscopy than reflected in the reported RCT results which also included prevalent cases that could no longer have been prevented by screening colonoscopy. Although these non-preventable CRC cases diminished reported screening effects, their earlier detection is an additional asset of screening as it enhances chances of cure. The presented patterns therefore imply the need for a more differentiated view on the evidence of the screening colonoscopy effects and may help to resolve some of the ongoing controversy regarding interpretation of the trial results [12] and some of the apparent discrepancy from findings from observational studies and real-life settings.

In the United States, CRC incidence has declined by approximately 50% in the screening age range in the past 30–40 years [13], despite adverse trends in the prevalence of key CRC risk factors such as obesity and increasing incidence at younger ages [14]. The most plausible explanation for this dramatic decline is the widespread use of colonoscopy for both screening and diagnostic purposes. With preventive effects of screening endoscopy in the order of magnitude of the reported NordICC trial results these real-life changes could not have been achieved. Our illustration may help to explain much of the apparent discrepancy between the reported RCT results and these real-life data.

Another factor to be considered in the interpretation of the RCT results is that diagnostic colonoscopies, which have similar preventive potential as screening endoscopies through detection and removal of CRC precursors, have meanwhile become more common also in European countries in which the NordICC trial was conducted [15]. Not accounting for diagnostic colonoscopies or, in general, colonoscopies outside the screening trials conducted during follow-up may further have attenuated the reported RCT effect estimates [16]. Whereas the relative importance of prevalence bias would be expected to gradually decrease with prolonged follow-up, such as 15- or 20-year follow-up of the trial cohort, the role of other biases such as contamination would be expected to further increase over time.

Although our numerical example focused on the first and so far only RCT results on the long-term impact of screening colonoscopy on CRC risk, the illustrated prevalence bias is expected to have similarly affected RCTs on CRC screening by other modalities, such as flexible sigmoidoscopy or fecal occult blood tests [2–6, 17, 18], whose preventive effects may likewise have been substantially underestimated. Although RCTs are less prone to various other biases than observational studies, the randomized design does not protect from prevalence bias which may be substantial in screening studies as illustrated in our article.

In summary, the preventive effects of screening endoscopy are likely to be stronger than suggested by the reported RCT results. Accounting for the prevalence bias leads to effect estimates that are much more in line with results from observational studies and real-life settings [13, 14, 19, 20]. More rigorous methodological work is needed to develop effective and user-friendly tools to prevent or adjust for prevalence bias in future screening studies. The apparently low RCT-based effect estimates should not unduly discourage use of CRC screening, the likely most effective way to cope with the ongoing global CRC epidemic, which is expected to lead to an increase in case numbers from approximately 1.9 million in 2020 to 3.2 million in 2040 [21]. In the contrary, efforts of prevention need to be enhanced, and major efforts are needed to better disentangle true prevention of CRC occurrence, early detection of already prevalent CRC, and their combined contribution to lowering the CRC burden in CRC screening studies.

## Acronyms and Abbreviations

CRC: colorectal cancer
ITS: intention-to-screen
NordICC: Nordic-European Initiative on Colorectal Cancer
PP: per-protocol
RCT: randomized controlled trial
